# Assessing client and staff beliefs and attitudes to inform tobacco-free campus policy implementation at substance use disorder treatment centers

**DOI:** 10.18332/tpc/202468

**Published:** 2025-03-24

**Authors:** Amogh Bandekar, Kim Bayha, Ashley Finke, Vanessa Mallory, Michael F. Dulin, Michael E. Thompson

**Affiliations:** 1 Stanford University Graduate School of Business, Stanford, United States; 2 Mecklenburg County Public Health, Charlotte, United States; 3 Premier Inc, Charlotte, United States; 4 Georgia State University, Atlanta, United States; 5 Department of Health Management and Policy and the Academy for Population Health Innovation, University of North Carolina at Charlotte, Charlotte, United States

**Keywords:** community-academic partnerships, preventative care, public health programs, COVID-19, tobacco cessation, health policy

## Abstract

**INTRODUCTION:**

US patients with behavioral health conditions have smoking rates two to three times higher than the general population. Tobacco-free environments at substance use disorder (SUD) facilities can positively impact patient’s outcomes as well as the health of staff, but client perceptions to the contrary can slow adoption. This study assessed client and staff beliefs, attitudes, and knowledge regarding the implementation of a tobacco-free campus policy at McLeod Addictive Disease Center, a full-service SUD treatment facility based in Charlotte, North Carolina.

**METHODS:**

During the height of the 2020 COVID-19 pandemic, the research team conducted a mixed-methods study at the McLeod Center lasting from May to November 2020. Using convenience sampling, the team conducted two staff surveys that were administered online (n=134; n=28) and virtual interviews of clients (n=38) to assess beliefs, attitudes and knowledge of tobacco use and the tobacco-free campus planned for 2021.

**RESULTS:**

Many staff identified as current or former smokers (n=57); some expressed the belief that the policy will positively impact clients’ SUD recovery (n=12). Encouragingly, clients expressed positive feelings associated with the policy (n=16) and reported interest in receiving tobacco cessation treatment (n=25).

**CONCLUSIONS:**

Staff are interested in helping clients quit tobacco use. Most of the interviewed clients, however, believed that utilizing tobacco products while receiving treatment for their dependence on another substance, would improve their success. The McLeod Center was one of the first community-based SUD treatment facilities in North Carolina to transition to a 100% tobacco-free campus in 2021. This research and results serve as a blueprint for other facilities making similar policy changes.

## INTRODUCTION

In the United States, patients with behavioral health (BH) conditions use tobacco at rates two to three times higher than the general population^1^. BH conditions include mental health or substance use disorders. BH patients who smoke will typically have a 17-year reduction in life expectancy^2^. As a result, these patients demonstrate higher death rates, increasing the cost and burden put on the healthcare system, and decreased quality of life and longevity. BH patients tend to display smoking behaviors at a young age and incur more difficulty when trying to quit smoking compared to the general population^3-6^. Past smoking interventions were less effective for BH patients. This shortcoming emphasizes the need for systemic policy-level interventions at behavioral health facilities and further study to demonstrate successful tobacco treatment interventions for this high-risk population.

The 5As (Ask, Advise, Assess, Assist, and Arrange) model of tobacco treatment is adequate and effective in treating tobacco use only when implemented correctly^7-9^. Among the 5As for tobacco treatment, assist and arrange follow-up are associated with increased quitting, but rates of assist and arrange follow-up tend to be low among healthcare providers^8^. Despite current treatment for tobacco usage (the 5As), research on adherence to these guidelines is scant. Current healthcare treatments are inadequate due to poor adherence to treatment guidelines. Staff within mental health and addictions facilities have a higher rate of smoking, approximately 10%, which is higher than general health practitioners whose smoking rate is 4%^10^. BH providers who are tobacco users are less successful in providing effective smoking cessation support for patients^11,12^.

Tobacco-free environments at substance use disorder (SUD) facilities have the potential to positively impact tobacco treatment; however, in 2016, fewer than half of SUD treatment facilities offered evidence-based tobacco cessation treatments^13^. Yet, policies around tobacco-free campuses (TFCs) can be effective in restaurants and worksites^14^, and smoking bans can result in positive effects on health inequalities^15,16^. However, inconsistent tobacco-free policies increase socio-economic inequalities already present among individuals who use tobacco^5,17,18^. Due to the lack of education and understanding of such policies, people are more likely not to abide by the voluntary rules and suggestions. When tobacco-free policies that enforce regulation in certain areas or environments are implemented, these policies help reduce certain inequalities^19,20^. The best interventions on tobacco use within a community have come from legislated and mandated tobacco-free policies in environments and increased tobacco product prices within the community^14,21,22^.

Tobacco-free campus policies effectively increase knowledge to address tobacco treatment among behavioral health workers^23^ and improve cessation outcomes^24,25^. Despite national guidelines addressing tobacco use in healthcare settings, smoking is often overlooked among SUD treatment facilities, partially because smoking is considered a lower priority in comparison to other substances and due to fear that quitting while in recovery may jeopardize sobriety^13,26^. However, a meta-analysis of 24 studies conducted between 2006 and 2016 determined that tobacco cessation treatment interventions often produced a positive impact or no impact on substance use outcomes^27^. In fact, another meta-analysis examining 19 smoking cessation interventions found a 25% increased likelihood of long-term abstinence from alcohol and illicit drugs among tobacco use interventions offered during substance use treatment^28^. In New York, a five-year evaluation of a statewide tobacco-free policy for SUD treatment facilities showed a decline in smoking prevalence among staff workers (35% in 2008 to 22% in 2013) and a decline in cigarettes smoked per day among clients (13.7 in 2008 to 10.2 in 2013) ^24^. Studies show that incorporating tobacco-related education sessions in conjunction with the adoption of TFC policy is effective in increasing knowledge to address tobacco treatment among behavioral health workers^16,23^.

Understanding outcomes related to integrating tobacco treatment and TFC policy implementation at substance use treatment centers can: 1) target populations in need; 2) improve access to services and delivery; 3) improve allocation of healthcare resources; and 4) reduce healthcare costs. This study assessed client and staff beliefs, attitudes, and knowledge regarding implementing a TFC policy at McLeod Addictive Disease Center, a full-service SUD treatment facility based in Charlotte, North Carolina.

## METHODS

This mixed-methods study was conducted at the McLeod Addictive Disease Center in Charlotte, North Carolina, from May to November 2020 to assess staff and client beliefs, attitudes, and knowledge of tobacco use and support for a TFC policy.

### Study population

The McLeod Addictive Disease Center is a community non-profit established in 1969 to provide quality behavioral health services to adults aged >18 years. It currently serves an average of 3900 patients daily who have a primary diagnosis of substance use disorder, in 9 locations across Piedmont and Western North Carolina. The facilities are in Charlotte, Concord, Gastonia, Hickory, Lenoir, Marion, Monroe, and Statesville. In 2020, McLeod served a total of 6599 unduplicated patients. Offering a menu of behavioral health services, McLeod employs a variety of staff ranging from case managers, nurses, substance abuse counselors/clinicians, management positions, data entry, and many more. The facilities offer their clients medication-assisted treatment services (MAT) while the main facility offers residential services, outpatient treatment, education programs, and case management.

In 2019, Mecklenburg County Public Health (MCPH) committed to addressing this health disparity and equity issue through the development of a Change for Life: Tobacco-Free Recovery Coalition of community-based BH agencies. McLeod Addictive Disease Center served as an early adopter pilot with a commitment to implement a TFC policy by January 2021, supported by tobacco-free communications and ongoing tobacco treatment integration.

### Measures

This mixed-methods study used two waves of a self-administered online convenience sample employee survey: May (n=134); August (n=28) and structured, virtual client interviews (n=38). The employee survey in May measured staff tobacco history, while the employee survey in August measured types of tobacco used and staff sentiments. Client interviews were conducted between May and November 2020. The study authors developed the interview guide and survey questions utilizing existing literature and input from the community partner, MCPH’s Tobacco Prevention & Control Program. The Tobacco Prevention & Control Program supervisor reviewed and edited the interview and survey guides.

### Eligibility criteria and recruitment

Participants were eligible for the study if they worked or were treated at the McLeod Center. Participants were excluded from the study if: 1) their primary language spoken was not English or Spanish; 2) they suffered from a severe cognitive impairment that limited their ability to participate; and 3) they had a life-threatening medical condition.

Participants were recruited for the online, self-administered employee survey through word of mouth, which included distributing a company-wide email sent from the president of the McLeod Center. Clients were recruited for the in-depth interview through signage and staff announcements in the facility.

Announcements informed potential respondents that participation was voluntary and that they would not be compensated for their time.

### Procedures

Employee responses were collected anonymously via SurveyMonkey. Survey questions focused on employee demographics, including current tobacco use status and employee sentiments and attitudes toward the TFC policy. These latter items included open-ended, free response questions.

Client interviews were conducted virtually due to safety constraints imposed by the ongoing COVID-19 pandemic. These virtual interviews required participants to access HD Meetings, the McLeod Center approved telehealth service, assuring compliance with the Health Insurance Portability and Accountability Act (HIPAA) and CFR 42 part 2. To initiate the virtual interview, clients entered a private room at one of the seven participating McLeod Center locations. Interviews lasted approximately 15 minutes and focused on their beliefs and attitudes toward the TFC policy after collecting demographic characteristics (age, gender, race/ethnicity, education level, and current tobacco status).

### Data analysis

The quantitative data were tabulated and summarized. Client interviews were transcribed. The transcripts were analyzed using grounded theory and thematic analysis. Two researchers reviewed the analysis and agreed on the coding and emergent themes. Preliminary findings were shared with the McLeod Center staff, administration, and clients for review and comment before finalizing the report (member checks).

## RESULTS

### McLeod Center employees

May survey

A total of 134 employees responded to the one-question survey administered in May 2020. Of them, 56% reported never smoking or having used tobacco products (including electronic cigarettes), 32% were former users, and 11% were current tobacco users.

*August survey*

The majority of the 28 employees who participated in the August survey had been employed with the McLeod Center longer than a year (84%), were female (74%), non-Hispanic White (81%), and had obtained a college degree (81%). The use of combustible tobacco products (cigarettes, cigars, cigarillos, pipe and/or hookah) was reported by 11% of employees and non-combustible tobacco products (e-cigarettes, electronic cigars, electronic cigarillos, electronic hookah, chewing tobacco, dip, snus, vaporizers, and/or vape pens or IQOS) was reported by 7% of employees. Secondhand smoke exposure while at work reportedly bothered 37% of employees.

When asked the question ‘If the McLeod Center continues to offer telehealth services after 1 January 2021, do you see any challenges to offering virtual tobacco use treatment?’, 15% of employees reported yes. Representative challenges included:

*‘We do need more tablets at the clinics. Sometimes one is insufficient, and we have clients waiting to use, especially with intakes.’*

*‘Although it would be a telehealth service, there could remain individuals who may want to take advantage of this treatment; to address their tobacco use if efforts improve health and save money. If the virtual tobacco treatment may not be attractive to all, however if we can benefit a portion of people then that is a plus.’*

*‘People will still smoke.’*

*‘Other tobacco users in the home.’*

The majority (64%) of employees believed clients would not be accepting of the TFC policy while also believing that the TFC policy would positively impact clients’ overall SUD recovery (64%); of note, however, 18% of employees did not believe the TFC policy would positively impact clients overall SUD recovery.

### Employee belief and attitudes around the TFC policy

Many employees expressed concerns and/or hesitation associated with the TFC policy, which included clients being resistant to change, frustrated with the policy, and clients potentially seeking services at other SUD organizations that did not have a TFC policy. Employees provided the following responses to their thoughts, suggestions and/or concerns with the policy:

*‘I think that it is good for us being viewed as a medical facility, but I feel that patients will continue to use tobacco when off campus.’*

*‘I worry about Anuvia [a competing communitybased treatment facility] not launching when we do, so clients who wish to smoke will choose to go there instead.’*

*‘It will be a deterrent for patients to utilize our services and they will seek treatment elsewhere. As a whole, trends are moving towards less restrictive attitudes in relation to substance use. In particular, use those substances without immediate and severe health impacts.’*

*‘There will likely be resistance as with any change.’*

*‘I believe it will place a lot of stress on the patients which will cause the patients to take their frustration out on staff.’*

*‘I believe that it will be highly ineffective, and employees will be tempted to sneak a smoke break in the bathrooms.’*

Several employees expressed positive sentiments towards the TFC policy, which included an overall benefit to employees and long-term SUD recovery for clients. These employees provided the following responses to their thoughts, suggestions and/or concerns with the policy:

*‘I believe the implementation of this policy is beneficial in assisting the clients in improving their opportunity for sobriety as many associate tobacco smoking with their drug use. I imagine there will be some, initial, discomfort for both staff and clients through the transitional period, however, this is an overall healthy measure to take.’*

*‘Smoking is the leading cause of preventable death in the US, causing over 480000 deaths per year. I’m glad we finally took a stand.’*

*‘I’m excited to be a part of McLeod Center at this important milestone, and I see the tobacco free policy as essential to the interests of staff, clients and the agency overall.’*

Employees frequently reported clear policy communications (n=6), employee and client participation (n=3), and employee support (n=2) to be salient factors in leading a successful policy implementation. Employees provided the following responses when addressing factors they believed necessary for successful policy implementation:

*‘Participation by all.’*

*‘If everyone participates and leads by example.’*

*‘A high percentage of buy-in from staff will be valuable, along with unwavering commitment from leadership to stay the course. It is essential that staff demonstrate empathy in their interactions with one another and with clients and their partners/families.’*

*‘Handouts to give clients, giving clients warnings, clear boundaries about where campus starts and ends.’*

*‘Making it clear to all staff and clients with signs.’*

### McLeod Center client interviews

The majority of the 38 clients interviewed were female (63%), aged <55 years (84%) and non-Hispanic White (92%), followed by non-Hispanic Black (8%) (Tables 1 and 2). The use of tobacco products, which included cigarettes, cigars, cigarillos, pipes, vapes, e-cigarettes, e-juice, JUUL, hookah, vape pens, dip, snuff, snus, and/or IQQS, was reported by 92% of clients. The majority (82%) of clients reported not being bothered by secondhand smoke while on the McLeod Center premises. Reassuringly, 74% of current tobacco users reported interest in receiving tobacco cessation treatment from the McLeod Center in the future.

**Table 1:** Characteristics of McLeod Center staff respondents, convenience sample, online survey, August 2020 (N=28)

*Characteristics*	*n*	*%*
**Employment duration** (years)		
<1	4	14.8
1–2	7	25.9
3–4	4	14.8
4–5	5	18.5
>5	7	25.9
**Biological sex**		
Male	5	18.5
Female	20	74.1
**Age** (years)		
18–24	1	3.9
25–34	5	19.2
35–44	7	26.9
45–54	8	30.8
55–64	5	19.2
**Race/ethnicity**		
Non-Hispanic White	22	81.5
Non-Hispanic Black	2	7.4
Hispanic	1	3.7
Other	0	0
**Education level**		
Some college	3	11.5
Associate’s degree	2	7.7
Bachelor’s degree	6	23.1
Graduate degree	15	57.7
**Combustible tobacco use**		
Yes	3	11.1
No	24	88.9
**Non-combustible tobacco use**		
Yes	2	7.1
No	26	92.9
**Bothered by secondhand smoke**		
Yes	10	37.0
No	17	63.0
**Virtual tobacco use treatment challenges**		
Yes	4	14.8
No	23	85.2
**TFC policy will positively impact client SUD recovery**		
Strongly agree	12	42.9
Agree	6	21.4
Neither agree nor disagree	5	17.9
Disagree	2	7.1
Strongly disagree	3	10.7
**Client acceptance of TFC policy**		
Strongly accepting	0	0
Accepting	5	17.9
Neither accepting nor not accepting	5	17.9
Not accepting	13	46.4
Strongly not accepting	5	17.9

TFC: tobacco-free campus. SUD: substance use disorder.

**Table 2:** Characteristics of McLeod Center clients completing in-depth interview, convenience sample, 2020 (N=38)

*Characteristics*	*n*	*%*
**Location site**		
Charlotte MAT	2	5.1
Charlotte Residential	8	20.5
Concord MAT	7	17.9
Hickory MAT	5	12.8
Lenoir MAT	8	20.5
Marion MAT	5	15.4
Monroe MAT	3	7.7
**Gender**		
Male	14	36.8
Female	24	63.2
**Age** (years)		
25–34	15	39.5
35–44	8	21.1
45–54	9	23.7
55–64	6	15.8
**Race/ethnicity**		
Non-Hispanic White	35	92.1
Non-Hispanic Black	3	7.9
**Education level**		
Lower than high school	8	21.1
High school graduate	17	44.7
Some college	9	23.7
College graduate	4	10.5
**Tobacco use**		
Yes	35	92.1
No	3	7.9
**Bothered by secondhand smoke**		
Yes	7	18.4
No	31	81.6
**Tobacco cessation treatment**		
Yes	25	73.5
No	9	26.5

MAT: medication-assisted treatment services.

### Client beliefs and attitudes around the TFC policy

Many clients expressed positive feelings toward the policy (n=16) when asked the question ‘How do you feel about the tobacco-free campus policy?’:

*‘I personally think it’s a really good idea, and the only reason I say that is because it is a treatment center. If you are a drug addict, and you are receiving treatment it is not beneficial to be smoking cigarettes with other drug addicts. You do not have to hang out at the McLeod Center and smoke cigarettes.’* (Concord; female, 25–34 years).

*‘To be honest with you, I was hoping that it was tobacco free before I came because I was going to try and quit smoking, so if no one else was smoking it would have made it easier.’* (Charlotte; male, 55–64 years).

Other clients expressed negative feelings associated with the policy (n=9) and the remaining clients either were not sure or expressed neutral feelings towards the policy (n=13):

*‘I don’t really care for it. I think that in my opinion if you know it’s a tobacco free campus there should be an area to smoke for people. I shouldn’t be judged or discriminated against for using tobacco.’* (Monroe; male, 25–34 years).

*‘I think it is going to be a negative thing because smoke is a stress reliever when coming off drugs. It’s going to be a bad thing right off hand, but people will get used to it. Smoking helps in stressful situations - yesterday I had grief counseling: my mom died and I cried all the time and smoked to relieve stress.’* (Charlotte; male, 35–44 years).

*‘Being a smoker, I don’t like it. I enjoy sitting outside and smoking to socialize.’* (Marion; female, 45–54 years).

Several clients provided suggestions and expressed concerns associated with the TFC policy when asked the question ‘Do you have any thoughts, suggestions or concerns about the tobacco-free campus policy?’. The most frequently provided suggestion consisted of incorporating a designated smoking area (n=6). Clients provided the following responses to thoughts, suggestions and/or concerns with the policy:

*‘I have the concern of causing more stress when people are in a vulnerable state. Cigarettes are an outlet - a breath of fresh air. That’s just my opinion.’* (Charlotte; male, 25–34 years)

*‘To be honest, I think it would work here only because it’s a small place so of course everybody was able to change with the COVID practices pretty quickly. Bigger campuses I’m not so sure.’* (Lenoir; female, 35–44 years)

Other respondents reported the implementation of a TFC policy would have a negative impact on their overall substance use recovery. Respondents provided the following responses for why they believed the TFC policy would have a negative impact on their long-term recovery:

*‘Smoking doesn’t have anything to do with my recovery’* (Monroe; female, 55–64 years)

*‘Will make things harder for people to get off drugs. I was on cocaine, others were on opioids, don’t want to hurt them. It’s going against the grain.’* (Charlotte; male, 35–44 years)

### Transitioning to a tobacco-free campus

Many clients provided suggestions for transitioning the McLeod Center to a tobacco-free campus. The most frequent response was policy communications (n=14), followed by monitoring and enforcing the policy (n=4), and incorporating a gradual implementation (n=3):

*‘Introduce it slowly; create a policy with phases.’* (Hickory; female, 25–34 years)

*‘If it’s not already, it needs to be posted in the lobby and on doors. I’ve known for years that you could smoke, so I came in knowing you could smoke. So it could be a problem for someone expecting to smoke on January 1.’* (Charlotte; female, 44–55 years).

*‘Be stern about the policy and put up signs about no tobacco use.’* (Monroe; male, 25–34 years).

## DISCUSSION

Overall, close to half of the sampled staff members were current or former smokers and were hesitant about the transition to a tobacco-free campus. Some sampled staff members expressed worries about clients choosing other facilities over the McLeod Center if other SUD treatment facilities in the area did not transition to becoming tobacco free. They believed that clients would be resistant, and that staff would bear the brunt of the inevitable client pushback on the policy.

The majority of the clients interviewed were unable to articulate a connection between tobacco use and their overall long-term substance use recovery. Many clients believed that utilizing tobacco products while simultaneously receiving treatment for their dependence on another substance would improve their ability to maintain abstinence. Clients were dismissive of their dependence on tobacco products because they rely on those products for stress relief and comfort when they are experiencing withdrawals from their primary substance dependence.

Other clients, however, indicated that transitioning to a tobacco-free campus would benefit their overall substance use recovery. They understand that utilizing tobacco during their treatment only increased their dependence on tobacco during the recovery process. These clients also realized that since they are receiving treatment for a substance use disorder, it is inconsistent to utilize tobacco products since they are also addictive.

Although the client’s responses varied regarding whether or not they supported the TFC policy, they made clear that the McLeod Center must be transparent during the implementation of this new policy. To minimize pushback, clients asked that they be informed on implementing the policy. Clients suggested creating appropriate signage and other communications for the center’s lobby and throughout the facility, and sending out messages via email or text reminders about the policy and future updates.

### Limitations

The convenience sampling methodology for the quantitative (employee) survey means the results might not represent the entire McLeod Center staff. In addition, these surveys study relied on self-reported data that can be subject to biases such as recall bias and social desirability bias. Due to the ongoing COVID-19 pandemic, the qualitative interviews were conducted virtually via HD Meeting, which is the telehealth platform utilized by the McLeod Center. At times during the interviews, the interviewer could not see the interviewee, and the connection occasionally lagged in response. Unstable connections sometimes caused the interviewee to have to repeat their answers to the interviewer and could have also resulted in the interviewer missing or misreporting the information provided. Interviewer bias was possible as they were aware of the interviewee’s smoking status. This knowledge may have resulted in the interviewer asking questions in a different tone and/or context to smokers than non-smokers. The stressful circumstances of the COVID-19 pandemic may have also influenced participants’ responses.

## CONCLUSIONS

Staff were aware of the benefits of tobacco cessation during clients’ SUD recovery; however, they remained hesitant about the center transitioning to a tobacco-free campus policy. Conversely, clients were unaware of the benefits of tobacco cessation towards their SUD recovery. With McLeod Center being one of the first community-based substance use disorder treatment facilities to transition to a tobacco-free campus, the research and results from this study could serve as a blueprint for other facilities that will be making similar policy changes. These facilities can review the information provided by clients and staff at the McLeod Center and implement these suggestions at their centers before they transition to tobacco-free. Future research should investigate how staff and clients perceive the now-implemented TFC policy^29^, how it is impacting clients’ overall substance use disorder recovery, and how it affects other physical health outcomes.

## Data Availability

The data supporting this research are available from the author on reasonable request.
